# A mixed methods exploration of the role of multi-family groups in community treatment of patients with depression and anxiety in Pakistan

**DOI:** 10.1186/s13033-021-00500-z

**Published:** 2021-10-19

**Authors:** Saniya Saleem, Anayat Baig, Sana Sajun, Victoria Bird, Stefan Priebe, Aneeta Pasha

**Affiliations:** 1 Q58254430 Interactive Research and Development, Karachi, Pakistan; 2 Unit for Social and Community Psychiatry, WHO Collaborating Centre for Mental Health Services Development, Queen Mary University of London, London, UK; 3 Global Health Directorate, Indus Hospital and Health Network, Karachi, Pakistan

**Keywords:** Psychosocial, Community-based, Common mental health disorders, Family involvement

## Abstract

**Background:**

An open, non-controlled trial was conducted to explore the feasibility, experiences and outcomes of multi-family groups in community mental health care of patients with depression and anxiety.

**Methods:**

The study was conducted in community settings within the catchment area of a free of cost primary care center in Karachi, Pakistan. 30 patients with symptoms of depression and anxiety, their caregivers and 3 lay counsellors were recruited enrolled in the study between May–September 2019. Patients were enrolled for monthly multi-family group meetings conducted over 6 months in groups of 5–6 patients and 1–2 nominated caregivers each. Meetings were facilitated by the non-specialist trained counsellors. The primary outcome was quality of life (assessed using Manchester Short Assessment of Quality of Life) and secondary outcomes were symptoms of depression and anxiety (assessed on Aga Khan University Depression and Anxiety Scale), social outcomes (Social Outcome Index), and caregiver burden (Burden Assessment Scale). Change in all measures was assessed pre and 6-month post intervention using t-test. In-depth interviews were conducted with 7 patients, 7 caregivers and the 3 lay counsellors.

**Results:**

A total of 36 family intervention meetings were conducted with six groups with a total of 30 patients, 34 caregivers and 3 counsellors. Between baseline and the end of the intervention, subjective quality of life increased significantly from 3.34 to 4.58 (p < 0.001, 95% CI 0.93–1.54). Self-reported depression and anxiety scores reduced from 34.7 to 19.5 (p < 0.001, 95% CI 10.8–19.8) and the Social Outcome Index improved from 3.63 to 4.52 (p < 0.001, 95% CI 0.39–1.39). There was no change in family burden. Participants reported that the group meetings were seen as a safe space for shared learning, and that the experience helped improve self-regulation of emotions and behaviors and instilled a sense of belonging.

**Conclusion:**

Multi-family groups in community treatment of common mental health disorders facilitated by non-specialist mental health service providers is feasible, experienced positively and has the potential for large and positive effects on subjective quality of life, self-reported depression and anxiety, and objective social outcomes.

*Trial Registration*: ISRCTN, ISRCTN12299326. Registered 05 June 2019. Retrospectively registered, https://doi.org/10.1186/ISRCTN12299326.

## Background

There has been a call to shift mental healthcare from institutions to community-based care in the Movement for Global Mental Health, with an emphasis on engagement with local communities [1, 2].This has led to families being given greater responsibility for support. Although stigma and discrimination against people living with mental illnesses are still prevalent [3] current mental health practices increasingly recognize the need to involve patients and their caregivers in their own care and management [4, 5].

Extensive research has explored different models for involving families in mental health care of patients with severe mental disorders, often focusing on psycho-education and various methods of family therapy [6]. Despite evidence to support involvement of families, family involvement in routine mental healthcare remains limited globally [7, 8]. There have been no known studies in the South Asian region to investigate the effectiveness of these approaches, specifically within people suffering from common mental health disorders.

In Pakistan, over 10% of the population (~20 million people) experience common mental health disorders including depression and anxiety with 70% of the population accessing out-of-pocket private healthcare [9]. Patients are mainly looked after by families [10], whilst training and family involvement in care delivery remain limited. The World Health Organization’s Mental Health Action Plan, 2013–2020, called for active involvement of people with mental disorders in the designing, planning and implementation of services to make care and treatment more responsive to their needs [4, 11].

A particular approach for involving families is the ‘trialogue’, a form of open communication in groups with professionals, patients and their families [12]. The multi-family groups in this study follow the principles of ‘trialogue’ and take components of a specific form of ‘trialogue’, called psychosis-seminars. Multi-family group in this study consist of five-six patients with one to two family member or friend each and a community mental health counsellor to chair the meeting. The approach emphasizes the civil rights and strengths of both patients and their families, requires mutual respect of all groups and promotes the sharing of experiences and learning within and across families and service providers [13–15].

This study aims to explore feasibility, experiences and outcomes of multi-family groups for people living with depression and anxiety in Karachi, Pakistan. In order to test a low-intensity intervention that can be implemented in low-resource settings, we planned to provide the intervention only once a month over a 6 month period [16]. Specifically, the objectives of this study are to determine:

1. how patients’ quality of life and other outcomes change during the 6-month intervention,
2. how the groups are experienced by participants, i.e. patients, family members/friends and mental health professionals, and.
3. whether multi-family groups for patients with anxiety and depression are feasible in the context of Pakistan.

## Methods

### Participants and setting

We conducted an open, non-controlled trial in community settings in Korangi, a low-income, urban district in Karachi, Pakistan. Patients visiting a primary care centre with integrated mental health service were assessed for depression and anxiety by a lay counsellor and enrolled for a brief intervention utilising a task-sharing approach. Lay counsellors were eligible to participate in the study if they had completed Interactive Research and Development Mental Health Program’s basic counselling training, had more than 6 months experience working with patients and did not plan to leave their position during the study period.

Patients visiting Indus Hospital and Health Network’s primary care center were approached to participate in the study if they met the following criteria: age between 18 and 65 years; resided within 20 km of the center, presented with symptoms of depression and anxiety (based on assessment on Aga Khan University Depression and Anxiety Scale (AKUADS)); and capacity to give informed consent. As the primary outcome was subjective quality of life, patients were excluded if they had a mean score of 5 or higher on the Manchester Short Assessment of Quality of Life (MANSA) scale. Consecutive patients were screened until enrollment was complete. Eligible patients completed a baseline questionnaire and were asked to identify two family members who were willing and interested in taking part in the study. ‘Family’ here is defined as anyone who is close with the patient and provides support. Consenting family members completed a short questionnaire including socio demographic information as well as a short assessment on caregiver burden.

Ethics approval was obtained by the Interactive Research Institutional Review Board (IRB 00005148) and Queen Mary University London Institutional Review Board (QMERC). The trial was retrospectively registered with the ISRCTN registry (ISRCTN12299326) on 5th June 2019.

### Materials and instruments

This was a mixed methods study and quantitative and qualitative data was collected. Basic demographics including age, gender, marital status, education status and employment status were collected for all participants.

The primary outcome for this study was subjective quality of life, measured on the MANSA. The MANSA comprises 16 items and patients rate their satisfaction on 12 life domains on a Likert scale from 1 to 7 (completely dissatisfied to completely satisfied). The mean score of these items is taken as indicator of subjective quality of life. The MANSA has been tested and used with patients in community settings [17].

Secondary outcome measures were the symptoms of depression and anxiety, and social outcomes. The AKUADS [18] is a locally developed and validated 25-item screening instrument for the symptoms of depression and anxiety. Based on a Likert scale rating of 0–3 for each question, scores range from 0 to 75, with higher scores indicating greater symptom severity. A score of 20 or above indicates probable depression and/or anxiety (Cronbach’s alpha = 0.69). The Social Outcome Index (SIX) [19] is an objective measure of social outcomes, designed to assess the employment, accommodation and social relationships of people living with mental illness. Scores range from 0 to 6, with higher scores indicating better social outcomes.

The Burden Assessment Scale (BAS) [20] 19 item questionnaire to assess objective and subjective caregiver burden was used to assess caregiver burden, with higher scores indicating higher burden (Cronbach’s alpha = 0.78).

A topic guide was developed for semi-structured interviews with participants in the local language, Urdu to learn about their experience with the intervention, barriers and facilitators to attending intervention sessions, suggested adaptations and the practical delivery of the intervention in the current context.

### Procedure

Patients were recruited between 27 May 2019 and 30 September 2019 and follow-up data was collected between December 2019 and January 2020. Written informed consent was obtained from all counsellors, patients and caregivers. All counsellors participating in the study received a 1-day training on group facilitation and implementation of the family involvement intervention.

The intervention involved monthly meetings of patients, their family members and counsellors to discuss pre-agreed, co-produced topics to allow learning through sharing experiences, mutual support and psychoeducation. At the end of each meeting, a meeting topic for the following meeting was agreed by mutual consensus of the group. Topics chosen by the groups were related to different life domains including relationship management, increasing self-awareness, the importance of physical health, recognizing signs of depression and anxiety, positive thinking, among others. The purpose of the meeting was to provide a safe space for patients and their family members to decide topics of concern, and benefit from communication, learnings, and psychoeducation from each other as well as the mental health facilitator. As such, the groups followed principles of mutual respect but allowed for flexibility so that group members could prioritize discussions around areas that concerned them. The meetings were held once per month over 6 months at local community centers and were chaired by trained counsellors. Each group consisted of five or six patients, one or two family members per patient, and the counsellor. Six multi-family groups were set up for the 6-month period, with each facilitator running two groups. Each family was reimbursed in local currency for travelling to the meeting site. A topic guide was developed in Urdu for semi-structured interviews with a subset of participant patients and service-providers after completion of intervention to learn about their experience with the intervention, barriers and facilitators to attending intervention sessions, suggested adaptations and the practical delivery of the intervention in the current context.

### Patient involvement

Patients and their families were not involved in setting the research question or the outcome measures, but they were they intimately involved in the co-development of the intervention design and implementation. Patients and family members along with the research team decided the agenda of each meeting.

### Data analysis

Quantitative data was analyzed using R software for data analysis. Descriptive statistics of demographic variables are provided with mean and standard deviation and frequencies for categories. Parametric analysis was conducted as assumptions of normality were met for the outcome variables of interest. Paired t-tests were used to assess changes in outcome measures over time. A p-value < 0.05 was considered statistically significant. Effect sizes were calculated for all outcome measures, using Cohen’s guidelines for effect size: 0.2 = small, 0.5 = medium, 0.8 = high [21].

Qualitative interviews were transcribed verbatim, and an inductive approach was used to provide new insights and richer understanding of the data without using preconceived categories. Two members of the research team (AB and SS) first familiarized themselves with the transcripts and used thematic analysis to examine the data, grouping similar under themes, and the identified themes and sub-themes discussed, checked, and refined by consensus. The transcripts were reviewed line by line by the first and second author independently, and text extraction was used to identify units of analysis and sorted into themes. Each theme was discussed and refined until consensus was reached. Direct quotations from the original transcript were included under each theme, maintaining the terminology used by the participants.

## Results

### Participants

**Fig. 1:**
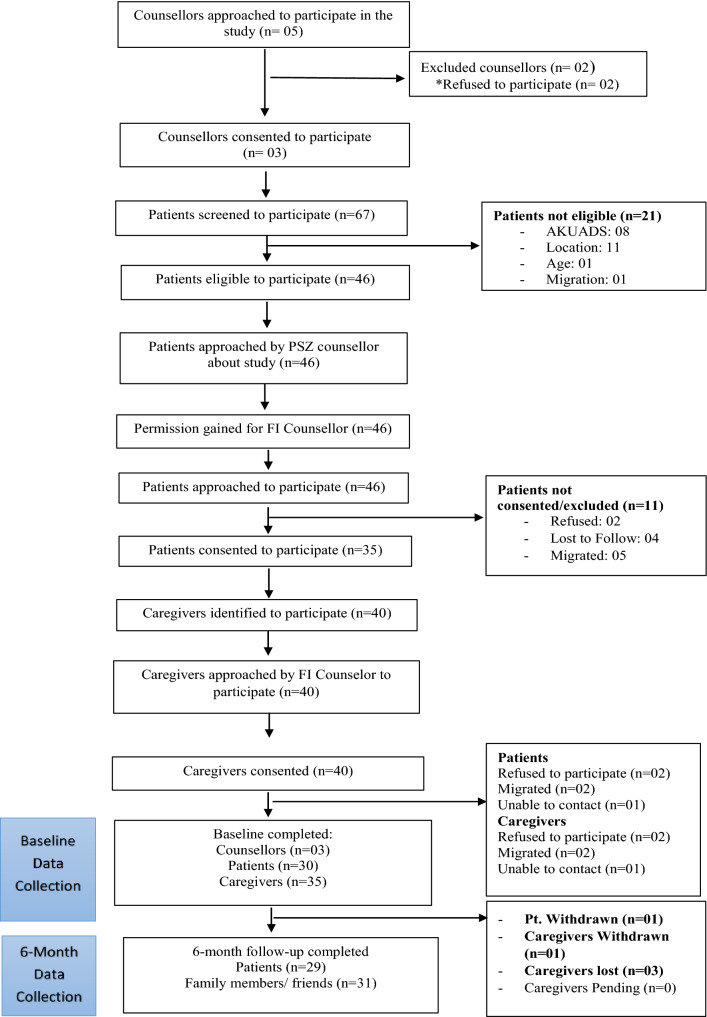
CONSORT diagram

A total of 5 counsellors and 67 patients were assessed for eligibility (Fig. 1). Following the screening, 3 counsellors and 30 patients and 35 of their caregivers were enrolled to participate in the study. Patients were predominantly female (23/30), and married (28/30) and the mean age of patients was 39 years (SD = 9.6 years). Most of the caregivers were immediate family members (spouse, parent/child) (29/34), and female (24/34). The mean age of caregivers was 35 years (SD = 12.0 years). All three of the counsellors were female.

The flow of participants through the study is shown in the CONSORT diagram (See Fig. 1).

### Intervention

A total of 36 family group sessions were conducted with 30 patients and 35 caregivers. Overall, patients in the intervention participated in an average of 4.5 sessions and caregivers participated in 3.6 sessions. 25 patients (83%) and 24 caregivers (71%) attended 3 or more sessions.

### Outcomes

Six-month assessments were completed for 29 of the 30 patients and 31 of the 34 caregivers. Paired comparisons of outcomes are presented in Table 1.

**Table 1:** Mean scores of subjective quality of life, anxiety and depression, and objective social outcomes pre and post intervention in patients

	Baseline (n = 30) M (SD)	6 months (n = 29) M (SD)	*t*	95% CI	Cohen’s d
Patients					
MANSA	3.34 (0.54)	4.58 (0.68)	8.04*	0.93 to 1.54	2.09
AKUADs	34.73 (8.1)	19.45 (9.1)	6.79*	10.8 to 19.8	1.78
SIX	3.63 (0.93)	4.52 (0.99)	3.54*	0.39 to 1.39	0.99
Caregivers					
Burden Assessment Scale	36.97 (9.83)	37.68 (8.32)	0.32	− 5.2 to 3.8	0.08

^*^p < *0.001*

All patient outcomes improved significantly during the intervention. The changes on each outcome reflect a large effect size. Family burden remained without significant changes.

### Experiences of patients, caregivers and counsellors

We conducted 17 individual in-depth interviews with participants (7 patients; 7 caregivers; 3 counsellors). Three themes emerged regarding the use of multi-family groups in the current setting: (1) Platform for shared learning (2) Self-regulation and (3) Sense of belonging. These themes are described in more detail with quotes for illustration.

### Platform for shared learning

Overall, the group was described as a space where diverse viewpoints were respected and seen as an opportunity to enhance learning about others as well as their own selves.

“The best thing was that we were people who came from different places and spoke about one issue and shared our views and learnt that what I was thinking was negative” [P, male, 32 y/o].

“I was very stressed in my mind, as if my mind was closed. And I often thought that if there was someone there to explain to me where I was wrong, what my illness is, what my shortcoming is, and if there is no shortcoming, how I can live better. In the discussion, I learnt about my own shortcoming as well that's why I never stopped going” [P, female, 38 y/o].

“At the meeting, they would ask about our qualities and I would go in thought that I don’t know my own strengths how can this be and I realized that there are strengths in me” [CG, female, 18 y/o].

While the groups were based on a premise of equal and shared ownership, there was some evidence that patients and caregivers viewed the counsellor as ‘experts’ and referred to the sessions as ‘classes’*.*

### Self-regulation

Participants felt that the experience had had an impact on their self-regulation of emotions and behaviours. These changes were also noted by caregivers and others around them.

“I would get very angry. I even tried to drink acid, cut myself, hurt myself in anger. But now I have learnt to control my anger, I haven’t tried to hurt myself again, I learnt this from the meetings. Now when I get angry I breathe deeply 3 times. I have power to control. Since the meetings there has been a lot of change in me, meaning I have learnt control within myself” [P, female, 18 y/o].

“Before I would talk like a ‘crazy person’, think about suicide, hit my children, but now it’s not like that, now I like everyone, I make them laugh, they stay happy. I am also interested in work, before I would not work. The talk got us out of the pain” [P, female, 50 y/o].

“(My daughter) would not go to relatives’ house before, now she has started going. Before she would not even ask me anything, now she takes care of me…presses my feet at night, gives me water, presses my clothes, oils my hair” [P, female, 50 y/o].

“Some people said that they would not even leave their house. From this (the meetings) their social skills improved, they said they gained confidence to talk. Females would say that they were very fearful in talking, through this platform they would talk openly” [C, female, 27 y/o].

### Sense of belonging

Participants described their experience of sharing with others as a source of strength and hope which allowed them to unburden and look forward.

“Seeing people there I felt, I was not alone, there were others too…It gave me strength, seeing other members there would encourage me” [P, female, 40 y/o].

“We would go in stress, and after we came back think how relaxed we have become, expressed ourselves. This should be there, patients feel relief, just medication does not do anything, talking is important” [CG, female, 30 y/o].

“(we) talked as if it was our own home, and we spoke openly, our relation with each other became like family members, we all knew what was going on with each other” [P, male, 38 y/o].

“I felt a connection, if there was a problem then it was everyone’s problem.” [C, female, 27 y/o].

*P = patient; CG = caregiver; C = counsellor.

## Discussion

This study explored the implementation of a specific form of family involvement as a resource-oriented intervention in community care of patients with anxiety and depression in Pakistan. Multi-family groups were facilitated by trained counsellors, provided only once per month over a 6-month period. The study showed very clear results. The groups were attended well. Patients’ subjective quality of life, symptom levels and objective social situation improved, and the effect sizes on each of these outcomes were large. Participants also reported that the group meetings were experienced as a safe space for shared learning, helped in self-regulation of emotions and behaviours and instilled a sense of belonging in the community.

Multi-family groups have traditionally been conducted with patients with serious mental illness [6], while insight into the role of family and community involvement for patients with common mental health disorders remain limited [22]. Partner involvement in this context has been assessed previously, a small scale study to assess partner involvement on depression outcomes in women found large effect post brief intervention (d = 0.72) [23]. To our knowledge, this is the first study conducted in Pakistan to assess an intervention of family involvement for improving outcomes for people with depression and anxiety. This study adds important insights on the acceptability and feasibility of family involvement for the management of common mental health disorders in resource-poor settings. Our findings support the principles of trialog that posit that outcomes improve if all three groups, patients, family members and service providers communicate openly on equal footing outside of traditional therapeutic environments such as clinical settings [24]. While some participants reported experiencing the meetings as ‘classes’, many saw this as a platform for shared learning. Participants in the study also reported experiencing the group as a large family and activating positive behaviours and social networks within the family and in their wider community. This is an important finding for low-resource settings where family dynamics and associated stigma of mental illness are often seen as exacerbating factors for the illness, and most patients are cared for at home by families [25].

Our qualitative findings suggest that family involvement can improve the relationship between patients and caregivers through greater understanding of mental health and needs of patients experiencing depression and anxiety. This is critical for low-resource settings since this level of engagement and knowledge sharing is entirely uncommon leaving patients and families with no access to such information [26]. Limited awareness around mental health contributes towards stigma, and it has been found that engagement with mental health service users and their experiences is a key ingredient in anti-stigma campaigns [27].

While our qualitative findings suggest improvement in the relationship between patients and carers, we found no improvement in perceived carer burden. This may be because patients with mild-moderate depression and anxiety do not necessarily identify as ‘patients in need’; however multi-family groups may have reinforced the identification with patient and carer roles and thus not reduced the burden of feeling responsibilities for each other [28]. Further, family members may experience being viewed as “resources for services”, especially if they are given more responsibility for providing care [5, 28]. Our study did not assess change in the mental health status of caregivers themselves pre and post intervention. Increased risk of depression and anxiety within caregivers of patients with serious mental illness has been reported [29, 30], particularly in lower socio-economic communities due to low availability of social support [31], however, research in caregivers for common mental health disorders remains limited.

Group facilitation by lay counsellors to deliver care was readily accepted by the group, similar to findings in other low-resource settings [32]. This may be attributable to the fact that counselors are typically from the same communities as the families, thus eliminating the power dynamic that is typical in traditional mental health care in some cultures. Further, this supports the sustainability of resource-oriented approaches in that group members could be trained to facilitate groups within their own community to continue to provide support at no cost. The COVID-19 pandemic, which further disrupted the pathways to already burdened mental healthcare, underlined the need for innovation in service delivery in the community [33]. Multi-family groups as provided in this study may be a flexible approach to widen the service provision at very low costs.

The effect sizes are all very large, an unusual finding for studies on psycho-social interventions and particularly remarkable considering the brief duration and low intensity of the intervention. The large improvements cannot be explained only by a drastically improved mood which may have influenced all ratings, since the Objective Social Index—observer rated and based on objective data such as employment and living situation—also improved markedly. Thus, all measured patient outcomes suggest a substantial and clinically relevant improvement during the intervention. Whilst this is most encouraging, one needs to take into account that there was no control group. Future studies with a randomized controlled design are warranted to support the effectiveness of the intervention compared to treatment as usual or to active control conditions. Further, assessing the processes through which the multi-family sessions impact mental health outcomes in low resource settings may help amplify the therapeutic value of the intervention.

## Conclusion

Routine service delivery for community treatment of common mental health disorders still focus on service delivery to the individual. The findings of this study can inform current practices in low resource settings with a focused approach on interventions which provide a range of service options available to patients in routine mental health care.

## Data Availability

The datasets used and analysed during the current study are available from the corresponding author (SS) on reasonable request.

## Abbreviations

MANSA: Manchester Short Assessment of Quality of Life
AKUADS: Aga Khan University Depression and Anxiety Scale
SIX: Social Outcome Index
BAS: Burden Assessment Scale
ISRCTN: International Standard Randomised Controlled Trial Number
